# Diagnostic pathology in 2012: development of digital pathology in an open access journal

**DOI:** 10.1186/1746-1596-8-3

**Published:** 2013-01-10

**Authors:** Klaus Kayser

**Affiliations:** 1 Charite, Homboldt University, Berlin, Germany

**Keywords:** Virtual slide, Open access publication, Interactive publication, Automated histomorphometry

## Abstract

**Abstract:**

Herein we describe and interpret the digital world of diagnostic surgical pathology, and take the in Pathology leading Open Access Journal *Diagnostic Pathology* as example.

**Virtual slide:**

http://www.diagnosticpathology.diagnomx.eu/vs/1944221953867351

## Background

The digital world, which is the digitalization of all kinds of data, forms the basics of communication. Communication is one of the main components in medical treatment and research. How does this condition reflect to scientific publication, especially to open access peer reviewed pathology journals?

### Virtual slides and virtual microscopy

Digitalization in surgical diagnostic pathology mainly involves partly and completely digitized images of microscopic slides (still images, virtual slides), the diagnostic work with these slides (virtual microscopy), measurements and derived clinical applications, as well as integration into Hospital Information System (HIS) and Laboratory Information Systems (LIS). Virtual Slides (VS) are quite large (about 3 GB) and demand a specific server. Our journal *Diagnostic Pathology* is the only one that integrates virtual slides in its articles to our knowledge.

### Open access journals

Peer reviewed open access journals are in some part equivalent, in other parts contrary to conventional peer reviewed scientific journals: Equivalent in terms of financial interest of the publisher, format of data/script presentation and reading, contrivers in the financial model. Authors who usually use grants have to pay, readers have free and unlimited access; articles are presented in conventional order such as summary, introduction, material and methods, etc. Additional features of the digitized environment such as “interactive publication” (similar to on-line scientific chat), performance of image analysis (readers might use open access available measurement systems such as EAMUS^TM^ (http://www.diagnomx.eu:8080/amp), or access to open structured atlases are not available at present to our knowledge.

### Diagnostic pathology in 2012

This Editorial written at the end of 2012 tries to present a survey of the journal’s development in 2012.

The journal *Diagnostic Pathology* started in 2006, and published 46 articles in that year. In 2011, the number of submitted articles accounted for 242. It will increase to more than 300 in 2012. 130 articles were published in 2011, with an average publication time of 73 days from submission. In 2012, more than 170 articles will be accepted for publication. Despite the potential for a negative influence on the citation index, the Impact Factor increased from 1.39 in 2011 to 1.64 in 2012.

The opportunity to publish virtual slides was offered in 2011. Several logistic constraints such as glass slide handling, uniform digitalization, registration of published VS, etc. had to overcome. Not all research articles are suitable for VS in contrast to case reports that significantly benefit from this technology. Until today, approximately 60 articles are provided with virtual slides, i.e., approximately 20% of all articles published in 2011 and 2012. In collaboration with the companies DiagnomX, Huron, Leica, and Motic three digitalization centers have been implemented for America (Huron), China and Asia (Motic), and Europe (Leica). Therefore, we expect that the percentage of articles provided with VS will significantly increase in 2013.

### Editor’s in Chief politics and perspectives

The journal *Diagnostic Pathology* publishes all kinds of articles that contribute to tissue-based diagnosis in general. These include classic medical diagnosis, research, training, education, new technologies and applications, innovative ideas and their implementation. The broad spectrum of accepted articles offers opportunities and contemporary limitations. It is in favour for the degree of popularity, and permits authors to publish research and other findings somewhat “out of their main interest or scientific spectrum”. Examples are the articles on *Schizophrenia proteomics* [(http://www.diagnosticpathology.org/content/1/1/1], published in 2006 and accessed by approximately 40,000 readers, *Evaluation of oxidative stress in diabetes mellitus* [http://www.diagnosticpathology.org/content/2/1/22], published in 2007 and accessed by approximately 11,000 readers, or the case report on a *Tubular adenoma* [http://www.diagnosticpathology.org/content/2/1/29], published in 2007 and accessed by approximately 20,000 readers. According to these data, case reports are not necessarily of less interest to readers than research or other articles.

The contemporary constraints include the negative influence on the impact factor, which means a strong boundary of scientific reputation. Journals with high impact factor are usually highly specialized, and some of them publish only a few manuscripts per year. At present, our journal *Diagnostic Pathology* ranges in the middle of all conventional and open access pathology journals ordered in accordance with the impact factor.

Our aim is to keep this balance between popularity and scientific reputation in general with some preference for popularity. Popularity is closely associated with innovation and assistance of science in developing countries, whereas scientific reputation is more related to the spread of already known or implemented research.

Tissue-based diagnosis and associated research are not limited to present structures and appearance of existent biology systems to our opinion. The biology environment is founded on the chemical characteristics of carbon which can be replaced by different atoms, especially silicon. Research on replacing carbon in living systems by silicon is in its childhood. We will certainly accept results and interpretation of such experiments for publication as well as those that deal with implantation of technical communicative devices in organs or living systems adding to the intelligence or behaviour of those animals and/or man.

In other words, our understanding of diagnostic pathology includes all expressions of future man made or natural life, even if they might sound science fiction. A specific ‘Hypothesis’ article type is already available for this type of study.

### Specific issues and gratitude

We can retrospectively look at a real successful year of *Diagnostic Pathology* although we were not able to implement all aims, we are still working to implement still outstanding issues such as interactive publication or automated pre-review procedures in order to diminish the period between submission and publication, and to give support for colleagues who still need formal and scientific advices for successful publication.

We will start optimistically into the New Year 2013. As usual, there are some changes necessary in the editorial board in order to keep the journal alive. Some colleagues will leave the board other will enter and hopefully will contribute to the journals future development as our leaving colleagues did. We are also very grateful to all colleagues who served as reviewers, advisors, and authors. We do thank our readers for their interest and appreciate all their comments, as well as our publication and production team for their support and advices.

We are aware that all of you, the author, the reader, our reviewer, our publication team and board member are the mandatory basis of all our aims, which are the development of medical research, the increase of understanding our nature and environment, and serving for an improved diagnosis and treatment of patients.

Please take our best wishes for a Blessed and Merry Christmas and a Happy, Healthy, and Fruitful New Year.

